# Higher yield sustainability and soil quality by reducing chemical fertilizer with organic fertilizer application under a single-cotton cropping system

**DOI:** 10.3389/fpls.2024.1494667

**Published:** 2024-11-14

**Authors:** Ning Wang, Jin Zhan, Keyun Feng, Jie Qi, Hongyu Nan

**Affiliations:** ^1^ Institute of Crop Sciences, Gansu Academy of Agricultural Sciences, Lanzhou, China; ^2^ College of Resources and Environment, University of Chinese Academy of Sciences, Beijing, China

**Keywords:** fertilization regimes, applied organic fertilizer, soil quality, cotton yield, sustainable productivity

## Abstract

The integrated application of chemical and organic fertilizers has been demonstrated to enhance soil fertility and the sustainable production of cotton yields. However, the impact of different fertilizer formulations on the sustainability of cotton production and soil quality over time have not been widely discussed. Here, we aimed to systematically evaluate the impact of different fertilization regimes [no fertilizer(CK), single application of chemical fertilizer(CF), 75% chemical fertilizer + 25% organic fertilizer (M1), 50% chemical fertilizer + 50% organic fertilizer (M2), 25% chemical fertilizer + 75% organic fertilizer (M3)] on soil quality, yield and yield sustainability in cotton fields in 2023 through a 10-year (2014-2023) field trial. Results showed that: (1) Compared to the natural state, different fertilization treatments significantly increased the average annual cotton yield and sustainable yield index (SYI) (*P*< 0.001), with the M1 treatment having the highest yield and the M2 treatment having the highest sustainable yield index (SYI). (2) Soil organic matter, soil total nitrogen, soil ammonium nitrogen, soil alkaline dissolved nitrogen, soil available phosphorus, and soil available potassium content showed the highest increase under the M1 treatment as compared to the natural state (*P*< 0.001). (3) Soil alkaline phosphatase enzyme activity was significantly increased by different fertilization treatments compared to the natural state (*P*< 0.05), M1, M2 and M3 treatments significantly increased soil urease enzyme activity and soil catalase enzyme activity (*P*< 0.001). (4) The random forest analysis showed that soil organic matter, soil nitrogen fractions (soil total nitrogen, soil ammonium nitrogen, soil alkali-hydrolyzable nitrogen, soil nitrate nitrogen), and available potassium content played a pivotal role in determining the yield and yield sustainability of cotton. (5) The highest soil quality index (SQI) value was observed in the M1. A markedly positive correlation was observed between the SQI and SYI (y = 0.03892x + 0.59609, R^2^ = 0.90379, *P* < 0.001), highlighting that the SQI constituted a significant factor in the sustainable production of cotton. These findings suggest that long-term application of chemical and organic fertilizers is an effective strategy for improving soil quality and cotton yield in continuous cropping while also contributing toward a more sustainable agricultural system.

## 1 Introduction

Cotton (*Gossypium hirsutum* L.) is the most significant oil- and fiber-producing crop globally (Li et al., 2022b). As a kind of field crop with strong stress resistance and wide adaptability, it plays an important role in global industrial and agricultural development (Constable and Bange, 2015). China is one of the world’s most significant producers and consumers of cotton, according to statistics from 1978 to 2021. Although cotton production increased 2.60 times (Feng et al., 2024), China’s cotton production remained insufficient in 2022, with an annual shortfall of approximately 1.62 million tons (Chen et al., 2023). Increasing the cotton planting area is difficult to boost yields because the cultivated land area is reduced and the benefit of cotton planting is limited. At the same time, long-term continuous cropping of cotton would cause a notable decline in soil quality and organic carbon content, which will affect cotton field yield and the environment (Tao et al., 2020; Adeli et al., 2022). It is therefore evident that the protection and enhancement of the quality of cotton fields is paramount for the assurance of sustainable cotton production and augmented yields. Furthermore, it is crucial for the guarantee of national food supplies.

The majority of studies have demonstrated that the enhancement of cotton yield is primarily attributable to genetic gain (Yang et al., 2020) and the optimization of agronomic management practices (Dai and Dong, 2014). A literature review on agronomic strategies for sustainable cotton production showed that key aspects include: (1) inoculation with arbuscular mycorrhizal species to enhance root exploration, biomass, and nutrient uptake; (2) using grass, legume, and brassica cover crops to reduce resource depletion; (3) adopting drip and mulched drip irrigation systems for water conservation; (4) exploring the feasibility of prematurely terminating irrigation in humid subtropical and Mediterranean climates as an alternative to chemical defoliation (Vitale et al., 2024). There is abundant scientific literature on the sustainable agronomic practices of cotton cultivation concerning fertilization, weed control, and proper water resource management (Vitale et al., 2024). Particularly, the integrated application of chemical fertilizers and organic manure is a well-established farming management practice with the proven ability to enhance crop yield and improve soil fertility (Chivenge et al., 2010; Diacono and Montemurro, 2010). In addition, crop yields are affected by the ratio of chemical fertilizers to organic fertilizers, climate and soil fertility conditions (Lu et al., 2018). Therefore, in exploring the effects of different organic and inorganic fertilizer formulations on crop yield, studies should be conducted in specific regions and specific cropping systems. In recent studies, crop yield stability has been employed as a metric for assessing the sustainability and resilience of agricultural production systems. The sustainable yield index (SYI) proved an invaluable tool for the assessment of the comparative sustainability of yield performance across a range of agricultural practices (de Albuquerque Nunes et al., 2021; Mi et al., 2023). Studies have focused on wheat (Han et al., 2020), millet (Yamoah et al., 2002), and rice (Mi et al., 2023) cropping systems, which are the major food crops under organic and inorganic fertilization, however, there is a relative paucity of research on sustainable production in cotton cropping systems.

Soil quality, as a concentration of maintaining crop productivity and protecting environmental quality (Doran and Parkin, 1996), can be evaluated by soil’s physical and chemical properties, biological indicators and nutrient content (Liu et al., 2006). Although studies have investigated changes in soil microbial biomass, enzyme activity (Wang et al., 2020) and nutrient content (Li et al., 2022a) in cotton fields under different fertilizer management practices, few kinds of literature have systematically evaluated the quality of soils in cotton cropping systems by integrating soil physical, chemical, and biological indices, and thus systematically. Furthermore, the response of individual soil attributes to different fertilization approaches may vary, thereby complicating the evaluation of soil quality (Raiesi and Kabiri, 2016). Therefore, it makes sense to integrate various soil parameters for specific soil properties into a single indicator for the whole. The soil quality index (SQI) is a valuable indicator for synthesizing a range of soil parameters into a unified measure that enhances understanding of soil function and processes (Obade and La, 2016). Indeed, there is a relative paucity of current work exploring the relationship between sustainable production and soil quality indice in single cotton continuous agroecosystems.

Gansu Hexi Corridor cotton planting area is an important part of the northwest inland cotton area and is a high-quality cotton production area in China (Fu et al., 2012). In recent years, to obtain higher yield benefits, the application of chemical fertilizers per unit area of cotton fields has been increasing, while the amount of organic fertilizers has been decreasing year by year, and the phenomenon of blind application of fertilizers has been more common (Feng et al., 2011), resulting in a series of problems such as declining soil fertility, nutrient imbalance and environmental pollution. Promoting the reduction of chemical fertilizer application through the efficient use of organic fertilizers to replace part of the chemical fertilizers is an effective way to alleviate the pressure of crop yield increase and environmental protection (Lu et al., 2018). This study investigated the effects of chemical fertilizer reduction and organic fertilizer application on soil physicochemical properties, soil nutrient content, soil enzyme activity, cotton yield and soil quality in the cotton growing area of the Hexi Corridor through a single cotton continuous agro-ecosystem location experiment lasting 10 years. This research will provide a theoretical foundation for the scientific fertilization of local cotton production and the prevention and control of environmental pollution. We hypothesized that (i) chemical fertilizers in combination with organic fertilizers have higher cotton yields, yield sustainability, and higher soil quality compared to the natural state and the use of chemical fertilizers alone. (ii) Soil organic matter and soil nitrogen fraction-related parameters could better predict cotton yield and sustainability.

## 2 Materials and methods

### 2.1 Research site

**Figure 1:**
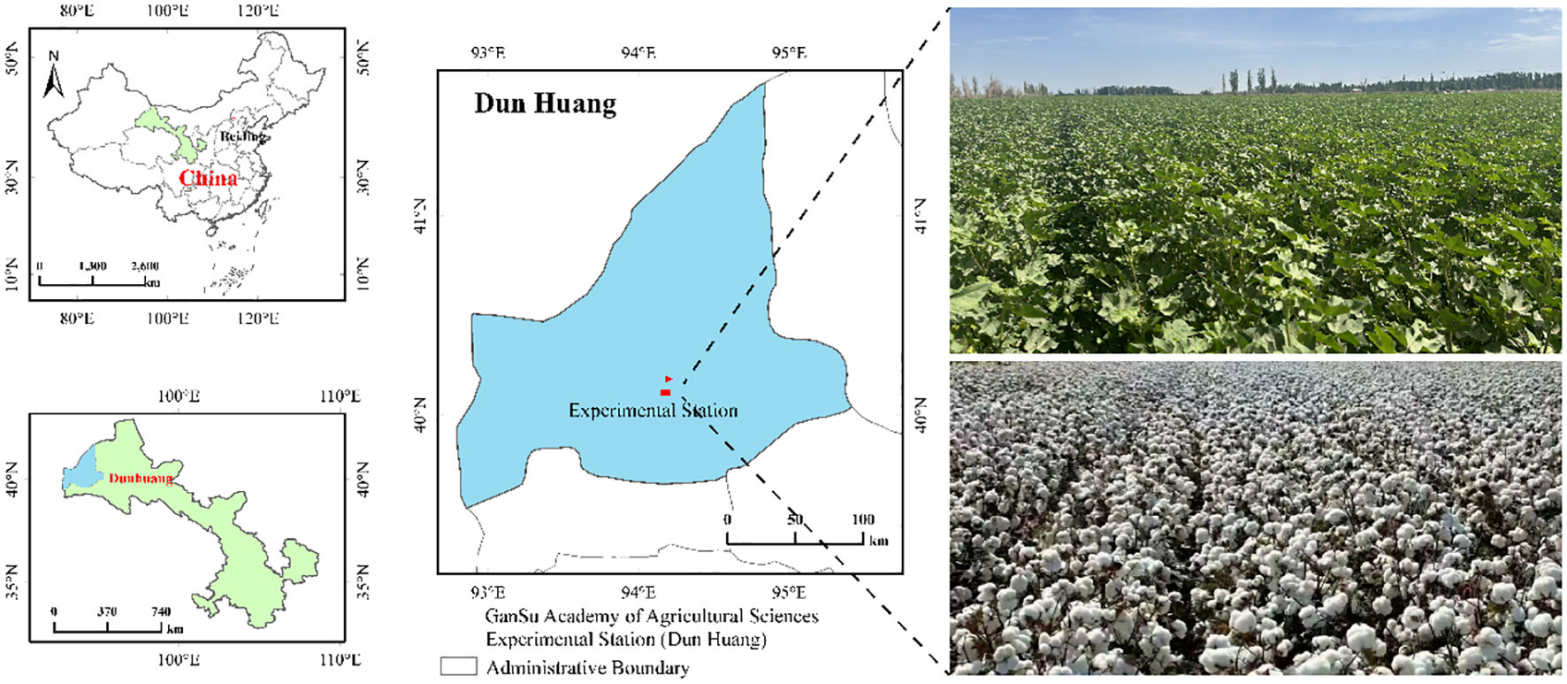
Schematic diagram of location of experimental area and cotton scene.

The long-term field experiment commenced in 2014 with a single cotton cropping system. The research site located in Dunhuang Cotton Experimental Station of Gansu Academy of Agricultural Sciences, Weijia Qiao village (40°01′ N, 94°38′ E), Dunhuang City, China (**Figure 1**). The station is located in the westernmost part of the Hexi Corridor, with an average elevation of 1138.00 m. The mean annual precipitation is about 39.90 mm, the evaporation is 2486.00 mm, and the average annual temperature is 10.50 °C, with a frost-free period of 145 d. The station has a typical continental arid climate. According to meteorological data (From 2014 to 2023, http://gs.cma.gov.cn), the variation in rainfall and temperature during the experiment is shown in **Supplementary Figure S1**. The soil of the experimental site was a silty soil with uniform fertility, containing 12.80 g·kg^-1^ of organic matter, 0.61 g·kg^-1^ of total nitrogen, 49.00 mg·kg^-1^ of alkali-hydrolyzable nitrogen, 28.30 mg·kg^-1^ of available phosphorus, 191.00 mg·kg^-1^ of available potassium, and soil pH was 7.30 (Wang et al., 2020).

### 2.2 Experimental design

The cotton variety subjected to testing was derived from the local staple variety, designated “Long Cotton No. 3,” which was selected by the Crop Research Institute of the Gansu Provincial Academy of Agricultural Sciences. The field experiment was conducted in a randomized block design with five treatments, six replications and a total of 30 plots. The area of each plot was 38.40 m^2^(8 m×4.8 m) and was planted under mulch covering, with 1 film and 4 rows, plant spacing of 15 cm, row spacing of 40 cm, and planting density of 165,000 plants ha^-1^. 2 m isolation zone was set up between each plot. Description of the five treatments was shown in **Table 1**, According to the amount of nitrogen fertilizer to calculate the fertilizer ratio, the cotton whole life cycle of nitrogen, phosphorus and potassium fertilizer application of all treatments to maintain the same amount of N 450.00 kg·ha^-1^, P_2_O_5_ 90.00 kg·ha^-1^, K_2_O 40.00 kg·ha^-1^, phosphorus and potassium content of the shortfall through the fertilizer to make up for it.

**Table 1:** Description of the treatment of fertilization regimes.

Treatment	Ratio of fertilizer and organic fertilizer
CK	0 fertilizer
CF	100% chemical fertilizer
M1	75% chemical fertilizer + 25% organic fertilizer
M2	50% chemical fertilizer + 50% organic fertilizer
M3	25% chemical fertilizer + 75% organic fertilizer

The organic fertilizer used in the experiment contains 45.00% organic matter, 7.20% total nitrogen, 2.60% total phosphorus, and 2.20% total potassium, in cotton before sowing as a base fertilizer one-time application. The test fertilizers were urea (N 46%), calcium superphosphate (P_2_O_5_ 11%), and potassium sulphate (K_2_O 50%). Phosphorus, potassium and 20% fertilizer nitrogen were used as the base fertilizer for each treatment in a one-time deep application before planting, and the remaining 80% of fertilizer nitrogen according to the cotton growth of fertilizer characteristics according to 25%, 25%, 25%, 25% ratio of 4 times with the irrigation water applied.

### 2.3 Sampling and analysis

Each year from 2014 to 2023, after cotton was matured, we took five representative plants from each plot, recorded the number of bolls per plant, boll weight per plant, and other yield components, and calculated yield based on the harvested seed cotton yield of the plot. Meanwhile, in 2023, we took samples from each plot using the ring-knife method to determine the soil bulk weight, and soil samples were collected at a depth of 0-10 cm using a soil drill with a diameter of 2.50 cm for subsequent physical and chemical analysis. The specific operation is as follows: in each treatment plot, five soil samples are to be collected, mixed into one portion on-site, passed through a 2.00 mm sieve to remove roots and stones, and then transported back to the laboratory for analysis.

The soil pH was determined using an E20-FiveEasy pH meter (Mettler Toledo, Giessen, Germany). The NO_3_^–^N concentration was quantified by photometrically at 220 nm and 275 nm {UV spectrophotometry (UV-1601, Shimadzu Inc.)}. The NH_4_^+^-N concentration was measured by the indophenol blue spectrophotometric method (UV-1601, Shimadzu Inc.) (Zhan et al., 2024). The alkali-hydrolyzed reduction diffusing method was employed for the evaluation of alkali-hydrolyzable nitrogen content in soil (Mi et al., 2018). The soil organic matter was quantified through oxidation with potassium dichromate, while the soil total nitrogen was determined by the indophenol blue colorimetric method (Xi et al., 2022). The soil available phosphorus was quantified by 0.5 mol/L NaHCO_3_ leaching-molybdenum blue colorimetric method, while soil available potassium was measured by NH_4_OAc leaching-flame photometric method (Li et al., 2022a).

The soil urease activity was quantified through the indophenol-blue colorimetry method (Guo et al., 2012). Soil catalase enzyme activity was determined by potassium permanganate titration, and the activity of the soil alkaline phosphatase enzyme was determined by a colorimetric method utilizing disodium benzene phosphate (Wang et al., 2020).

### 2.4 Calculations

A comprehensive assessment of the soil quality of cotton land under different fertilization treatments over 10 years was carried out by calculating the Soil Quality Index (SQI). First, each soil factor data was converted to a score value (*Si*) between 0 and 1.0. Based on the fact that soil factors can be categorized into two types: “the more the better” and “the less the better”, if the soil factor is positively correlated with soil quality, it is scored using formula 1 for “the more the better”; On the contrary, the “less is better” formula 2 is used for scoring.


(1) Si=(Xi−Xmin)/(Xmax−Xmin)

(2) Si=(Xmax−Xi)/(Xmax−Xmin)

In this context, the term “ *Si*” represents the score of the *i*th factor, while “*Xi*,” “*Xmax*,” and “*Xmin*” correspond to the measured, maximum, and minimum values of the *i*th indicator, respectively (Chang et al., 2023; Li et al., 2024; Tian et al., 2024).

Secondly, the 12 soil factors were subjected to principal component analysis with the objective of extracting the common factor variance of each factor. Subsequently, the weight (*Wi*) of the *i*th factor was calculated based on the ratio of the common factor variance of the *i*th factor to the sum of the common factor variances of all factors (Li et al., 2019). Finally, the soil quality index (SQI) was determined through the application of formula 3, whereby elevated SQI values are indicative of superior soil quality.


(3) $$SQI=\sum_{i=1}^{n}WiSi$$

Where SQI is the soil quality index, *Wi* is the weight value of each factor, *Si* is the factor score, and n is the number of factors (Chen et al., 2021; Guo et al., 2024; Tian et al., 2024).

In order to assess the sustainability of cotton yield, the sustainable yield index (SYI) was employed (Mi et al., 2023). The formula is as follows:


SYI = (Ymean−σ)/Ymax

In this context, Ymean represents the mean yield of the treatment, σ denotes the standard deviation, and Ymax signifies the highest yield observed for each treatment over time. According to the aforementioned formula, if the standard deviation is low and the mean yield is high, the SYI will be high, indicating the potential for sustainable production under the specified management practice (Wanjari et al., 2004).

### 2.5 Statistical analysis

The distribution of all parameters was evaluated for normality and homogeneity of variance using the Shapiro-Wilk test and Levene’s test, respectively. One-way ANOVA was employed to ascertain the significance of the impact of disparate fertilization treatments on cotton grain yield, soil enzyme activities, and the physical and chemical properties of the soil. Significant differences were identified at the 0.05 level of significance.

The pearson correlation test was employed to examine the interrelationship between the soil quality index, mean yield, and soil environmental indicators. Then, to further analyze the factors affecting soil quality index and cotton mean yield, the random forest analysis was used to evaluate the importance of each soil environmental factor. Finally, in order to investigate the relationship between soil quality index and cotton grain yield or sustainable yield index, a linear regression analysis was conducted.

Data analyses were carried out employing IBM SPSS Statistics 22.0 (IBM Inc., Armonk, USA). The random forest analysis was carried out by the “randomForest”, “rfPermute” and “A3” packages of R version 4.2.2. The figures were generated using OriginPro 2021 (Origin Lab Corp., Northampton, USA).

## 3 Results

### 3.1 Soil physical and chemical indicators

**Figure 2:**
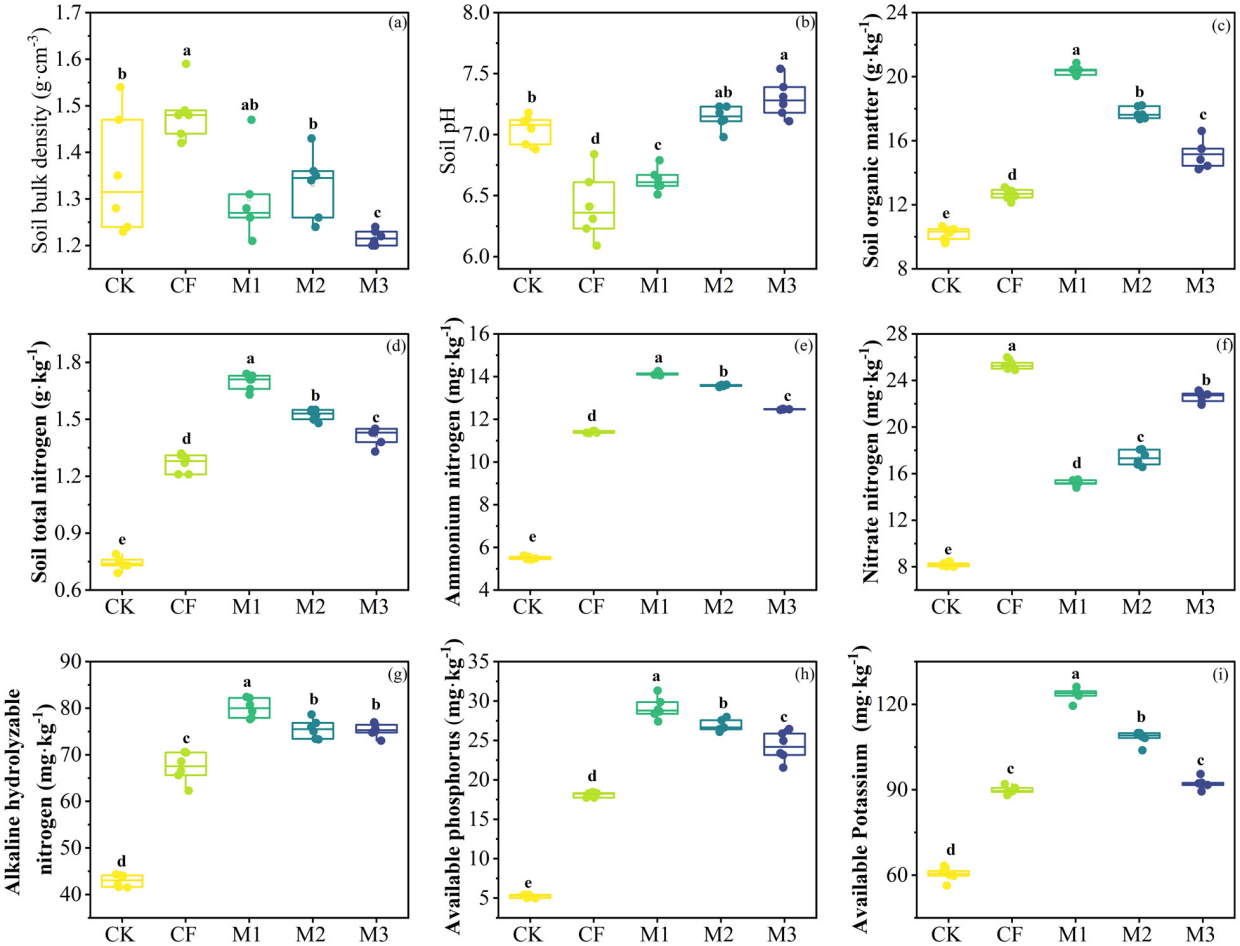
Soil physical and chemical indicators after 10 years of different fertilization practices. **(A)** Soil bulk density. **(B)** Soil pH. **(C)** Soil organic matter. **(D)** Soil total nitrogen. **(E)** Ammonium nitrogen. **(F)** Nitrate nitrogen. **(G)** Alkaline hydrolysable nitrogen. **(H)** Available phosphorus. **(I)** Available Potassium. (mean ± *SE*). Different lowercase letters indicated significant differences between treatments (*P*<0.05). CK: no fertilizer; CF: single application of chemical fertilizer; M1: 75% chemical fertilizer + 25% organic fertilizer; M2: 50% chemical fertilizer + 50% organic fertilizer; M3: 25% chemical fertilizer + 75% organic fertilizer.

The combined application of different fertilization treatments had an effect on topsoil physical and chemical indicators (**Table 2**). Compared to the CK, CF significantly increased soil bulk density by 9.74% (*P*< 0.01; **Figure 2A**), CF and M1 largely decreased soil pH (*P*< 0.05; **Figure 2B**). M3 significantly reduced soil bulk density from 1.35 g·cm^-3^ to 1.22 g·cm^-3^ (*P*< 0.01; **Figure 2A**), and elevated soil pH from 7.04 to 7.30 (*P*< 0.05; **Figure 2B**). Then, different fertilization treatments significantly increased soil organic matter, soil total nitrogen and soil ammonium nitrogen, with the highest increase in the M1 treatment, which resulted in 99.56%, 90.33%,156.35%, respectively (*P*< 0.001; **Figures 2C–E**). At the same time, different fertilization treatments significantly elevated soil nitrate nitrogen, soil alkali-hydrolyzable nitrogen, soil available phosphorus and soil available potassium, the CF treatment resulted in the highest increase in soil nitrate nitrogen, increasing it from 8.18 mg·kg^-1^ to 25.32 mg·kg^-1^ (*P*< 0.001; **Figure 2F**), the M1 treatment resulted in the highest increase in soil alkali-hydrolyzable nitrogen, soil available phosphorus and soil available potassium content in the soil (*P*< 0.001; **Figures 2G–I**).

**Table 2:** Summary of ANOVA for soil environmental indicators, cotton mean yield, soil quality index and sustainable yield index under different fertilization practices.

Index	df	*F*-value	*P*-value
BD	4	8.524	0.000
pH	4	31.425	0.000
SOM	4	379.241	0.000
TN	4	472.739	0.000
NH_4_^+^-N	4	24566.902	0.000
NO_3_^-^-N	4	1526.635	0.000
Alkali-N	4	294.703	0.000
AP	4	461.262	0.000
AK	4	786.890	0.000
Ur	4	122.520	0.000
CAT	4	111.949	0.000
ALP	4	6.574	0.001
Mean yield	4	77452.661	0.000
SQI	4	1135.519	0.000
SYI	4	331003.329	0.000

BD, soil bulk density; SOM, soil organic matter; TN, soil total nitrogen; NH_4_^+^-N, soil ammonium nitrogen; NO_3_^-^-N, soil nitrate nitrogen; Alkali-N, soil alkali-hydrolyzable nitrogen; AP, soil available phosphorus; AK, soil available potassium; Ur, soil urease enzyme activity; CAT, soil catalase enzyme activity; ALP, soil alkaline phosphatase enzyme activity; SYI, sustainable yield index; SQI, soil quality index.

### 3.2 Soil enzyme activities

**Figure 3:**
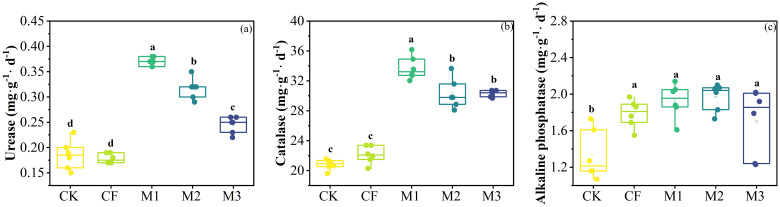
Soil enzyme activities after 10 years of different fertilization practices. **(A)** Urease. **(B)** Catalase. **(C)** Alkaline phosphatase. (mean ± *SE*). Different lowercase letters indicated significant differences between treatments (*P*<0.05). CK: no fertilizer; CF: single application of chemical fertilizer; M1: 75% chemical fertilizer + 25% organic fertilizer; M2: 50% chemical fertilizer + 50% organic fertilizer; M3: 25% chemical fertilizer + 75% organic fertilizer.

Compared to the CK, M1, M2 and M3 significantly increased soil urease enzyme activity by 100.00%, 71.17%, 32.43%, respectively (*P*< 0.001; **Figure 3A**), enhanced soil catalase enzyme activity by 62.06%, 45.62%, 45.63%, respectively (*P*< 0.001; **Figure 3B**). The different fertilization treatments significantly increased soil alkaline phosphatase enzyme activity (*P*< 0.05; **Figure 3C**).

### 3.3 Relations between soil indicators and cotton grain yield

**Figure 4:**
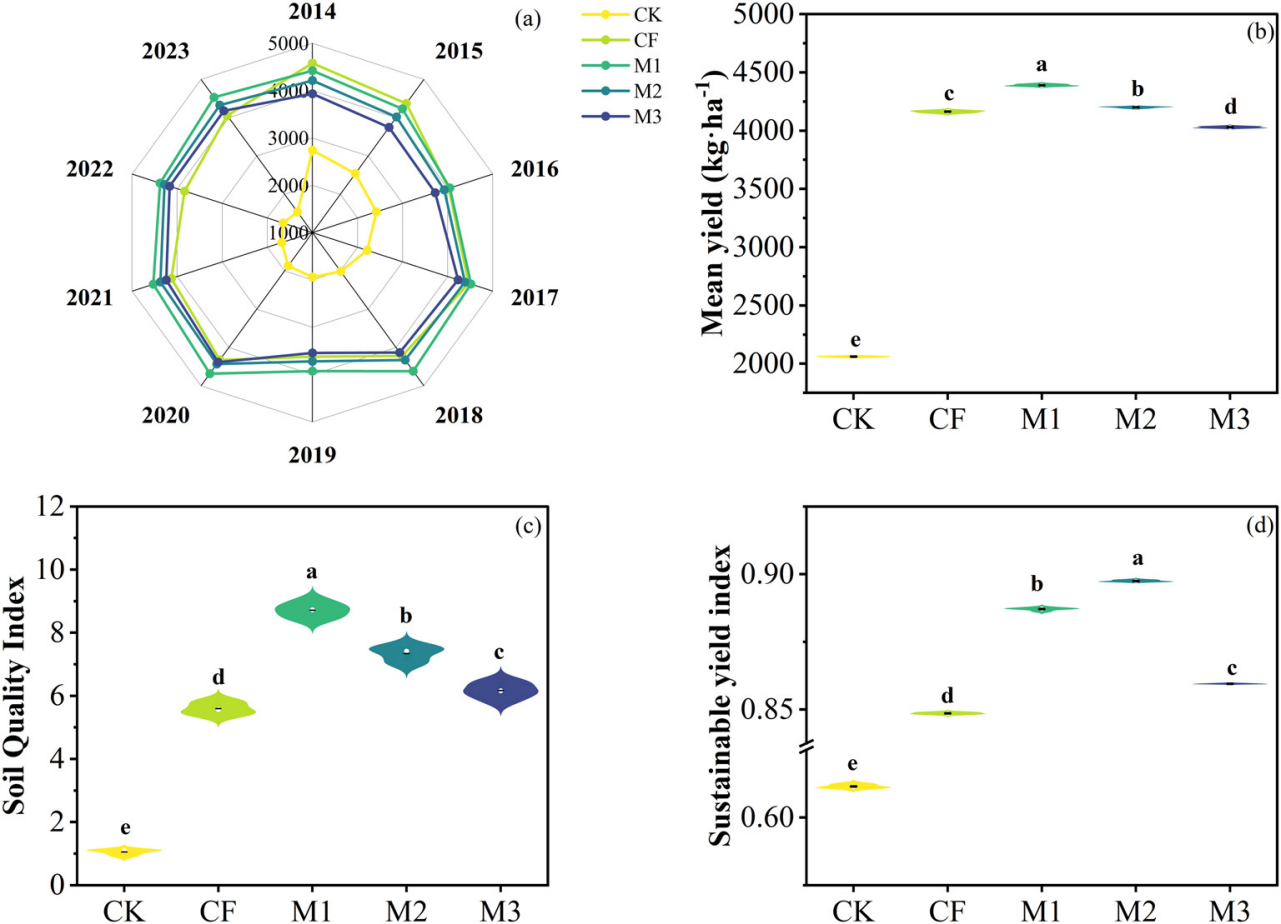
Cotton grain yield (kg ha^-1^) from 2014 to 2023 **(A)**, mean yield **(B)**, soil quality index **(C)** and sustainable yield index **(D)** under different fertilization practices. Different lowercase letters indicated significant differences between treatments (*P*<0.05). CK: no fertilizer; CF: single application of chemical fertilizer; M1: 75% chemical fertilizer + 25% organic fertilizer; M2: 50% chemical fertilizer + 50% organic fertilizer; M3: 25% chemical fertilizer + 75% organic fertilizer.

From 2014 to 2023, cotton grain yield in its natural state (CK) showed a decreasing trend over year (**Figure 4A**). Compared to the CK, the different fertilization treatments all facilitated cotton grain yield, with the highest increase in the M1 treatment (**Figure 4A**). The results of ANOVA for average annual cotton grain yield showed that different fertilizer treatments significantly increased cotton yield compared to the natural state, with the highest increase in M1 treatment (**Table 2**, *P*< 0.001; **Figure 4B**). The calculated sustainable yield index (SYI) showed a positive response to different fertilization treatments, and M2 treatment was the highest followed by M1(*P*< 0.001; **Figure 4D**).

**Figure 5:**
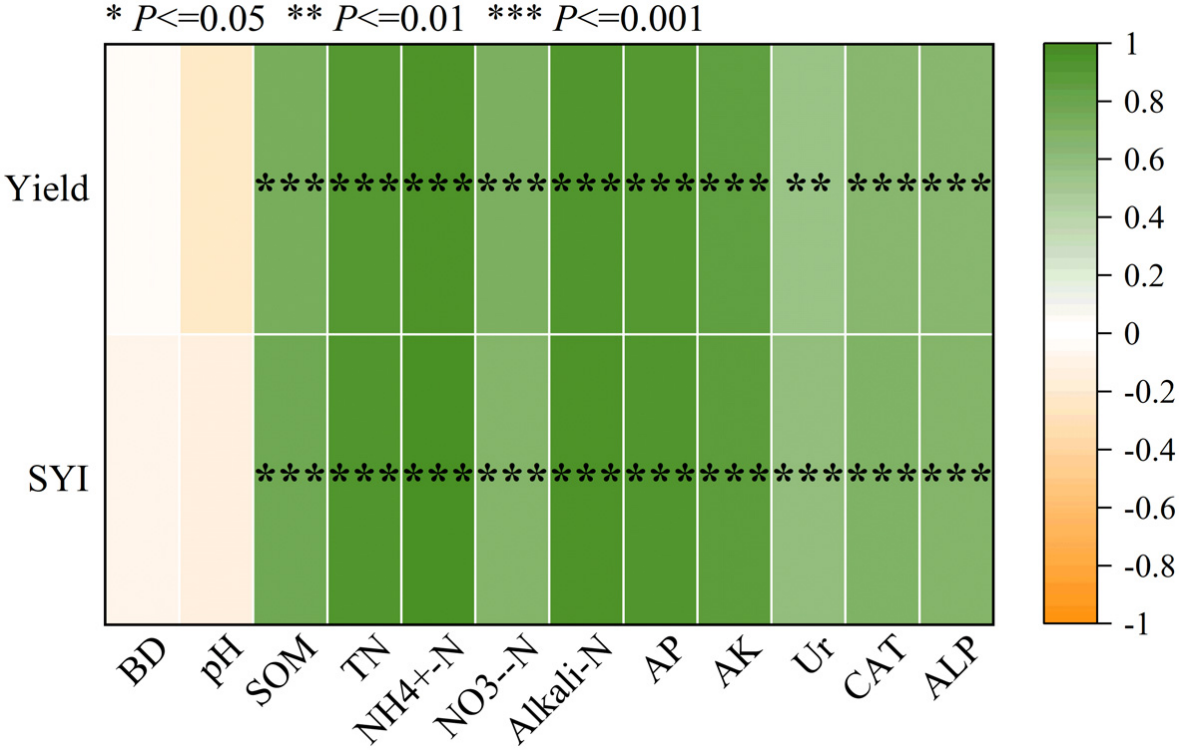
Pearson correlation coefficient between soil quality index, mean yield and soil environmental indicators. The asterisks indicate significant correlation coefficients (***P<0.001; **P < 0.01; *P < 0.05). BD, soil bulk density; SOM, soil organic matter; TN, soil total nitrogen; NH_4_^+^-N, soil ammonium nitrogen; NO_3_^-^-N, soil nitrate nitrogen; Alkali-N, soil alkali-hydrolyzable nitrogen; AP, soil available phosphorus; AK, soil available potassium; Ur, soil urease enzyme activity; CAT, soil catalase enzyme activity; ALP, soil alkaline phosphatase enzyme activity; SYI, sustainable yield index; SQI, soil quality index.

In general, cotton grain yield and sustainable yield index were positively associated with soil organic matter (SOM), soil total nitrogen (TN), soil ammonium nitrogen (NH_4_^+^-N), soil nitrate nitrogen (NO_3_^–^N), soil alkali-hydrolyzable nitrogen (Alkali-N), soil available phosphorus (AP), soil available potassium (AK), soil urease enzyme activity (Ur), soil catalase enzyme activity (CAT), and soil alkaline phosphatase enzyme activity (ALP) (*P <* 0.01; **Figure 5**). There was no significant correlation between soil bulk density (BD) and soil pH(*P>* 0.05; **Figure 5**).

**Figure 6:**
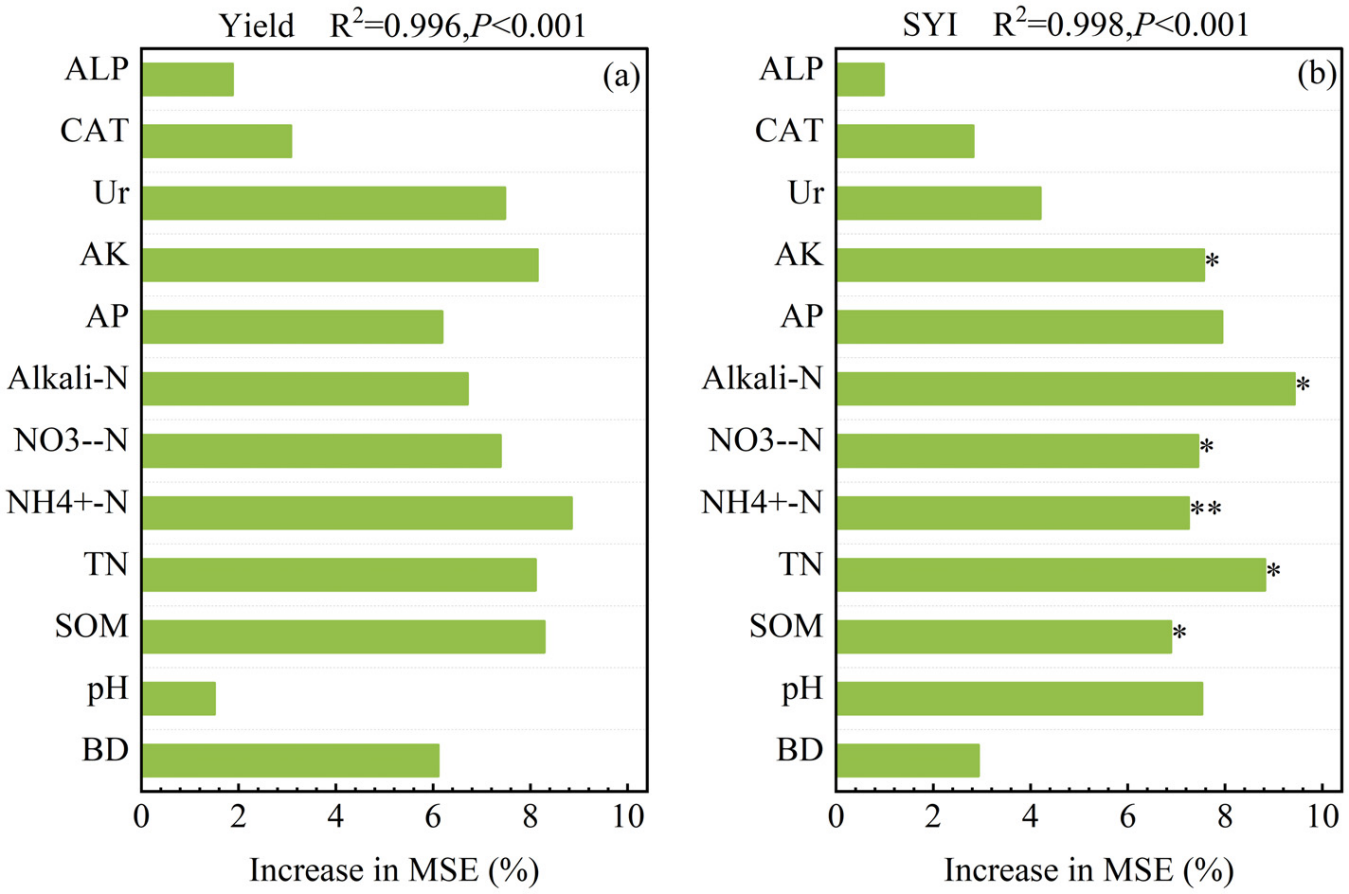
**(A, B)** The random forest analysis between soil quality index, mean yield and soil environmental indicators. The asterisks indicate significant differences between soil indicators (**P < 0.01; *P < 0.05). BD, soil bulk density; SOM, soil organic matter; TN, soil total nitrogen; NH_4_^+^-N, soil ammonium nitrogen; NO_3_^-^-N, soil nitrate nitrogen; Alkali-N, soil alkali-hydrolyzable nitrogen; AP, soil available phosphorus; AK, soil available potassium; Ur, soil urease enzyme activity; CAT, soil catalase enzyme activity; ALP, soil alkaline phosphatase enzyme activity; SYI, sustainable yield index; SQI, soil quality index.

The random forest analysis showed that twelve soil indicators accounted for 99.60% of cotton grain yield to different fertilization managements, and soil ammonium nitrogen, soil organic matter, soil available potassium (AK) and soil total nitrogen (TN) were the most important indicators followed by soil nitrate nitrogen (NO_3_^–^N) and Soil urease enzyme activity (Ur) (**Figure 6A**). At the same time, the random forest analysis showed that twelve soil indicators accounted for 99.80% of cotton sustainable yield index to different fertilization managements, while soil available potassium, soil alkali-hydrolyzable nitrogen, soil nitrate nitrogen, soil ammonium nitrogen, soil total nitrogen and soil organic matter were the most important indicators (**Figure 6B**).

### 3.4 Soil quality index

**Figure 7:**
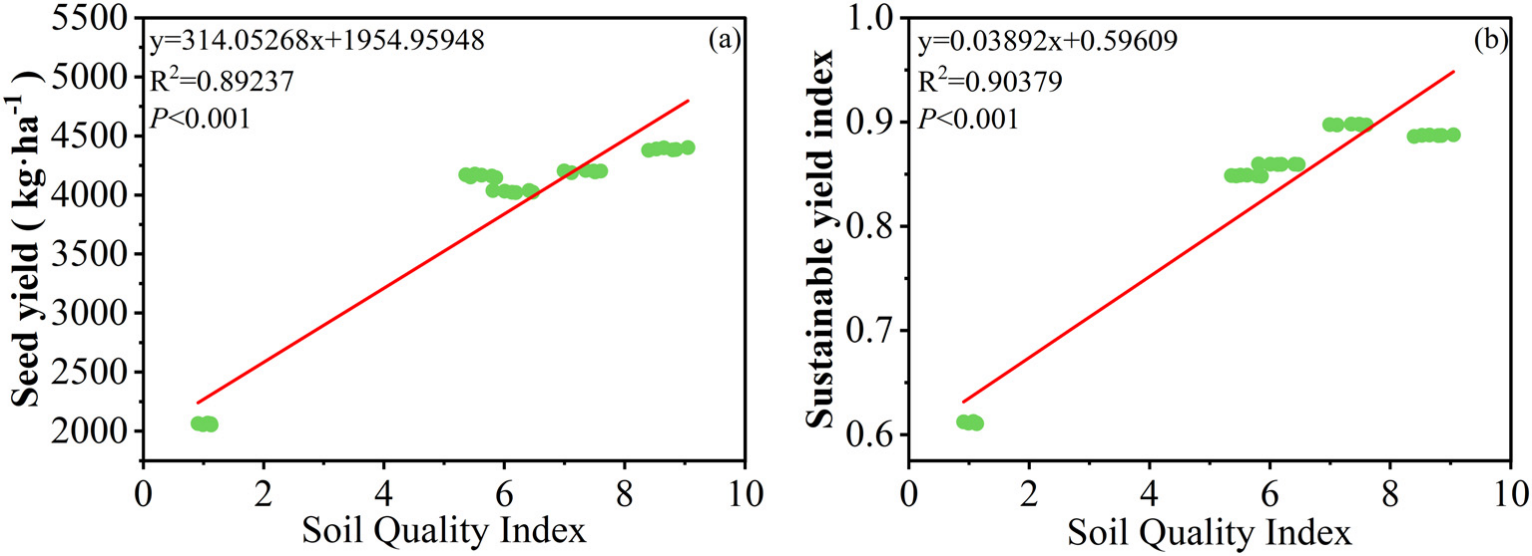
Relationships between soil quality index and cotton grain yield **(A)** or sustainable yield index **(B)**.

Soil quality index (SQI) values were calculated, ranging from 1.05 to 8.71 (**Figure 4C**). Compared to the CK, the different fertilization treatments all significantly increased soil quality index, with the highest increase in the M1 treatment (*P*< 0.001; **Figure 4C**). The SQI demonstrated a robust positive correlation with both cotton grain yield and the sustainable yield index (SYI), thereby substantiating the assertion that the soil quality index constitutes a pivotal contributor to sustainable cotton production (**Figures 7A, B**).

## 4 Discussion

### 4.1 Effects of different fertilization treatments on the cotton yield

Organic and inorganic fertilizer blending can reduce nutrient loss from farmland (Liu et al., 2017), improve fertilizer utilization (Lu et al., 2018), increase soil organic matter content, activate soil nutrients, improve the number and structure of soil microbial communities, change the activity of inter-root soil enzymes, alleviate the occurrence of soil salinization and soil-borne diseases, improve crop root vigor, antioxidant enzyme activity, and leaf pigmentation, and ultimately increase crop yields (Yamoah et al., 2002; Chivenge et al., 2010; Song et al., 2018). Shi et al. observed that the chemical fertilizers with organic fertilizers could improve soil organic matter content and nutrient effectiveness, optimize nutrient absorption and distribution by cotton plants, and improve seed cotton yield and nutrient utilization efficiency through two years of field trials (Shi et al., 2024). Lu found in a locational fertilization trial lasting 3 years of continuous cropping in cotton fields that substituting partial chemical fertilizer with commercial organic fertilizer was an economically viable, environmentally sustainable, and practical approach for enhancing soil nutrients, promoting cotton growth, and maintaining high and stable yield (Lu et al., 2021). Ma observed in continuous cropping of cotton fields for six consecutive years that continuous application of nitrogen fertilizer in conjunction with biochar resulted in enhanced nitrogen use efficiency and yield of cotton, as well as the promotion of N fertilizer transformation into the soil organic nitrogen pool (Ma et al., 2023). Cotton yield in the natural state in this study decreased yearly from 2014 to 2023, whereas different fertilization treatments significantly increased the average annual cotton yield, and chemical fertilizers and organic fertilizers had higher cotton yield. This suggests that rational fertilization improves the quality of cotton field soil, which in turn improves the yield of cotton fields. Organic fertilizers release nutrients slowly and provide crops with the nutrients they need for a long time, while chemical fertilizers are fast-acting and easily absorbed and utilized by crops. Chemical fertilizers and organic fertilizers can regulate the release rate and intensity of soil and fertilizer nutrients, improve the nutrient supply of the soil, which is more conducive to crop yield and stable production (Zhao et al., 2016; Yang et al., 2018). As in this study, different fertilization treatments significantly promoted the release of soil organic matter, soil total nitrogen, soil ammonium nitrogen, soil nitrate nitrogen, soil alkali-hydrolyzable nitrogen, soil available phosphorus and soil available potassium contents, which increased the soil quality index(SQI), and the calculated sustainable yield index(SYI) was positively affected by different fertilization treatments. At the same time, chemical fertilizers and organic fertilizers had higher soil quality index and cotton sustainable yield index. This verified hypothesis 1 of this study. The 75% chemical fertilizer with 25% organic fertilizer treatment was the most effective in increasing the average annual cotton yield among the chemical fertilizer reduction with organic fertilizer application in this study. The result indicated that under 75% chemical fertilizer with 25% organic fertilizer, the nutrient release pattern of the fertilizer matches the nutrient demand pattern of cotton growth (Wang et al., 2020). The highest increase in soil enzyme (urease enzyme and catalase enzyme) activities was observed in cotton plots under 75% chemical fertilizer with 25% organic fertilizer treatment. Higher soil enzyme activity reflects the vigor of substance metabolism in the soil (Zhang et al., 2018), which accelerates the nutrient transformation in cotton soil, resulting in a significant increase in soil nutrients such as soil organic matter, soil total nitrogen, soil ammonium nitrogen, soil alkali-hydrolyzable nitrogen, soil available phosphorus, soil available potassium, resulting in efficient utilization of nutrients and consequently the highest yield. As in this study 75% chemical fertilizer with 25% organic fertilizer treatment had the best improvement in soil quality index.

The sustainability of crop production represents a significant aspect of the broader sustainability of agricultural systems (Chaudhury et al., 2005; Xie et al., 2016). The study found that the highest SYI (System Yield Index) was recorded with the treatment of 50% chemical fertilizer and 50% organic fertilizer, indicating the application of 50% chemical fertilizer with 50% organic fertilizer demonstrated a favorable impact on the consistent production of cotton yields in a single-cotton cropping system. The observed outcome may be attributed to the long-term sustainability of cotton yield under a fertilization regimen comprising 50% chemical fertilizer and 50% organic fertilizer. This is because the continuous supply of nutrients during the vegetative growth of cotton may be a contributing factor to the observed result. On the other hand, the application of equal amounts of organic and chemical fertilizers improves the quality of the soil, thus increasing the resistance of cotton production to climate change. A study has demonstrated that the application of reduced quantities of inorganic fertilizer in conjunction with organic fertilizer can result in enhanced crop production and greater resilience to climate change (Qiao et al., 2022). Furthermore, the 75% chemical fertilizer with 25% organic fertilizer treatment demonstrated the most pronounced improvement in soil quality index among the treatments evaluated in this study. However, the SYI was not the highest under the 75% chemical fertilizer with 25% organic fertilizer treatment, ranking second only to the 50% chemical fertilizer with 50% organic fertilizer treatment. This may be due to the fact that a low standard deviation of yield coupled with a high mean yield, as indicated by Wanjari (Wanjari et al., 2004), leads to a high SYI, suggesting sustainable production under this management practice. The average annual cotton field yields for the lasting ten years in this study were highest under the 75% chemical fertilizer with 25% organic fertilizer treatment, followed by 50% chemical fertilizer with 50% organic fertilizer, and the magnitude of change in cotton field yields for the last ten years was relatively stable under the 50% chemical fertilizer with 50% organic fertilizer treatment. Whereas, the yield of a cotton field in 2019 under 75% chemical fertilizer with 25% organic fertilizer treatment decreased abruptly and drastically, which made the SYI under 75% chemical fertilizer with 25% organic fertilizer treatment lower than that of 50% chemical fertilizer with 50% organic fertilizer treatment.

### 4.2 Relationship of soil indicators with cotton yield

The increase in crop yields from chemical fertilizers with organic fertilizers is a combined effect related to the increased nutrient effectiveness of organic fertilizers, stimulation of crop growth and improvement of soil physical and chemical properties (Lu et al., 2018). In addition, crop yield was found to be significantly correlated with soil nutrients, microorganisms, and related enzyme activities. Furthermore, a significant correlation was observed between soil nutrients, microorganisms, and soil enzyme activities (Zhao et al., 2011; Jiang et al., 2017), which together contributed to the formation of crop yield. In the present study, cotton yield and sustainable yield index were positively associated with soil organic matter, soil total nitrogen, soil ammonium nitrogen, soil nitrate nitrogen, soil alkali-hydrolyzable nitrogen, soil available phosphorus, soil available potassium, soil urease enzyme activity, soil catalase enzyme activity, and soil alkaline phosphatase enzyme activity, and showed a highly significant positive correlation with soil quality index. Also, the random forest analysis showed that 12 soil indicators explained 99.60% and 99.80% of the variation in cotton yield and sustained yield index, respectively. Soil organic matter, soil nitrogen fractions (soil total nitrogen, soil ammonium nitrogen, soil alkali-hydrolyzable nitrogen, soil nitrate nitrogen) and soil available potassium were important factors influencing cotton yield and sustained yield index. This verified hypothesis 2 of this study. The result may be explained by the fact that firstly, as the basis for crop growth and development, the amount of major nutrients in the soil reflects the ability of the soil to provide and harmonize nutrients for crop growth (Ilyas et al., 2019; Meng et al., 2024). Among them, soil organic matter is the basis for securing nutrient supply (Bin et al., 2022), and the amount of total nitrogen and soil alkali-hydrolyzable nitrogen are key indicators of soil fertility and nutrient status (Li et al., 2022a). More than 50% of nitrogen nutrient uptake by plants comes from ammonium and nitrate nitrogen in the soil (Wu et al., 2018). Ammonium and nitrate nitrogen can be directly absorbed by plant roots, promoting root growth, while accelerating the absorption of phosphorus and potassium and other nutrients, increasing the number and area of leaves, and thus improving photosynthesis (Wang et al., 2019; Fu et al., 2023). Secondly, soil available potassium plays an important role in crop yield formation as a massive mineral nutrient essential for crop growth and development (Xue et al., 2019). Available potassium facilitates plant nutrient uptake and transport, is an activator of a number of enzymes in photosynthesis, and enhances stress tolerance, which affects crop yield and quality (Wang and Wu, 2017; Johnson et al., 2022). A study has shown that fertilization can affect the content of nitrate nitrogen and ammonium nitrogen in soil to meet the nitrogen demand during the growth and development of cotton, and then regulate the content of potassium in soil to promote the accumulation of dry matter of cotton and realize the increase of cotton yield (Meng et al., 2024). Therefore, soil organic matter, soil nitrogen fractions (soil total nitrogen, soil ammonium nitrogen, soil alkali-hydrolyzable nitrogen, soil nitrate nitrogen), and available potassium content are important indicators affecting the yield and yield sustainability of cotton.

## 5 Conclusions

The 75% chemical fertilizer with 25% organic fertilizer had a greater positive effect on cotton yield and soil quality index compared to the other fertilization treatments. The 50% chemical fertilizer with 50% organic fertilizer was more favorable for the sustainability of cotton yield. Soil organic matter, soil nitrogen fractions (soil total nitrogen, soil ammonium nitrogen, soil alkali-hydrolyzable nitrogen, soil nitrate nitrogen), and available potassium content were crucial soil indicators affecting the yield and yield sustainability of cotton. The soil quality index constituted a pivotal element in the advancement of sustainable cotton production.

## Data Availability

The original contributions presented in the study are included in the article/**Supplementary Material**. Further inquiries can be directed to the corresponding author.
