# Supplementary material for: Higher yield sustainability and soil quality by reducing chemical fertilizer with organic fertilizer application under a single-cotton cropping system
Source: Front Plant Sci. 2024 Nov 14;15:1494667. doi: 10.3389/fpls.2024.1494667 (PMC11602747; doi:10.3389/fpls.2024.1494667)
Supplement: Supplementary file 1 [file DataSheet1.docx]

Figure S1 Rainfall and average temperature in experimental area


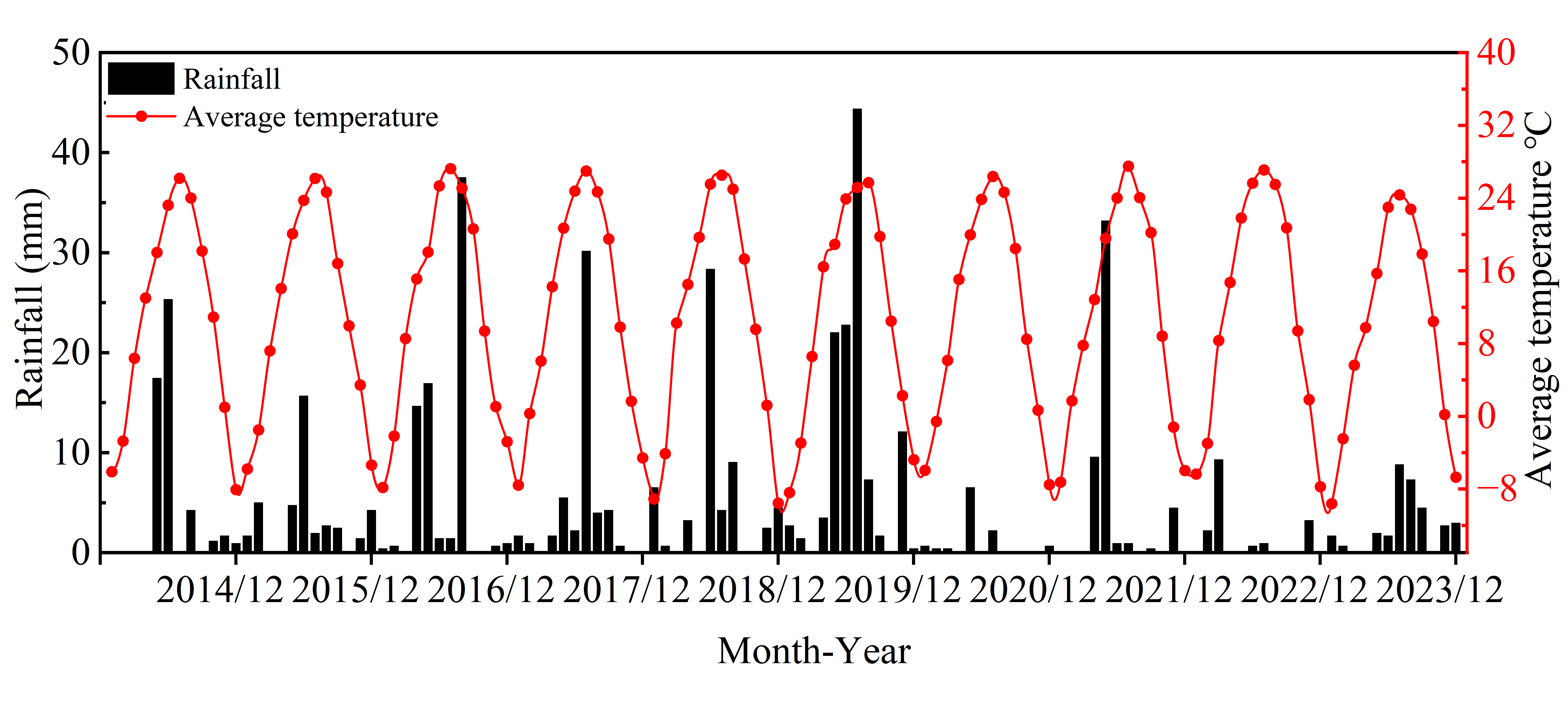


Table S1 Common factor variance of indicators extracted from principal component analysis of soil indicators. BD, soil bulk density; SOM, soil organic matter; TN, soil total nitrogen; NH_4_^+^-N, soil ammonium nitrogen; NO_3_^-^-N, soil nitrate nitrogen; Alkali-N, soil alkali-hydrolyzable nitrogen; AP, soil available phosphorus; AK, soil available potassium; Ur, soil urease enzyme activity; CAT, soil catalase enzyme activity; ALP, soil alkaline phosphatase enzyme activity; SYI, sustainable yield index; SQI, soil quality index.

| Index | Common factor variance |
| --- | --- |
| BD | 0.544 |
| pH | 0.833 |
| SOM | 0.976 |
| TN | 0.976 |
| NH_4_^+^-N | 0.990 |
| NO_3_^-^-N | 0.954 |
| Alkali-N | 0.967 |
| AP | 0.980 |
| AK | 0.952 |
| Ur | 0.956 |
| CAT | 0.894 |
| ALP | 0.530 |
